# Supplementary material for: Phosphatase protector alpha4 (α4) is involved in adipocyte maintenance and mitochondrial homeostasis through regulation of insulin signaling
Source: Nat Commun. 2022 Oct 14;13:6092. doi: 10.1038/s41467-022-33842-4 (PMC9568526; doi:10.1038/s41467-022-33842-4)
Supplement: Supplementary file 1 — Supplementary Information [file 41467_2022_33842_MOESM1_ESM.pdf]

Supplementary Information

Masaji Sakaguchi, Shota Okagawa, Yuma Okubo, Yuri Otsuka, Kazuki Fukuda, Motoyuki Igata, Tatsuya Kondo, Yoshifumi Sato, Tatsuya Yoshizawa, Takaichi Fukuda, Kazuya Yamagata, Weikang Cai, Yu-Hua Tseng, Nobuo Sakaguchi, C. Ronald Kahn and Eiichi Araki.

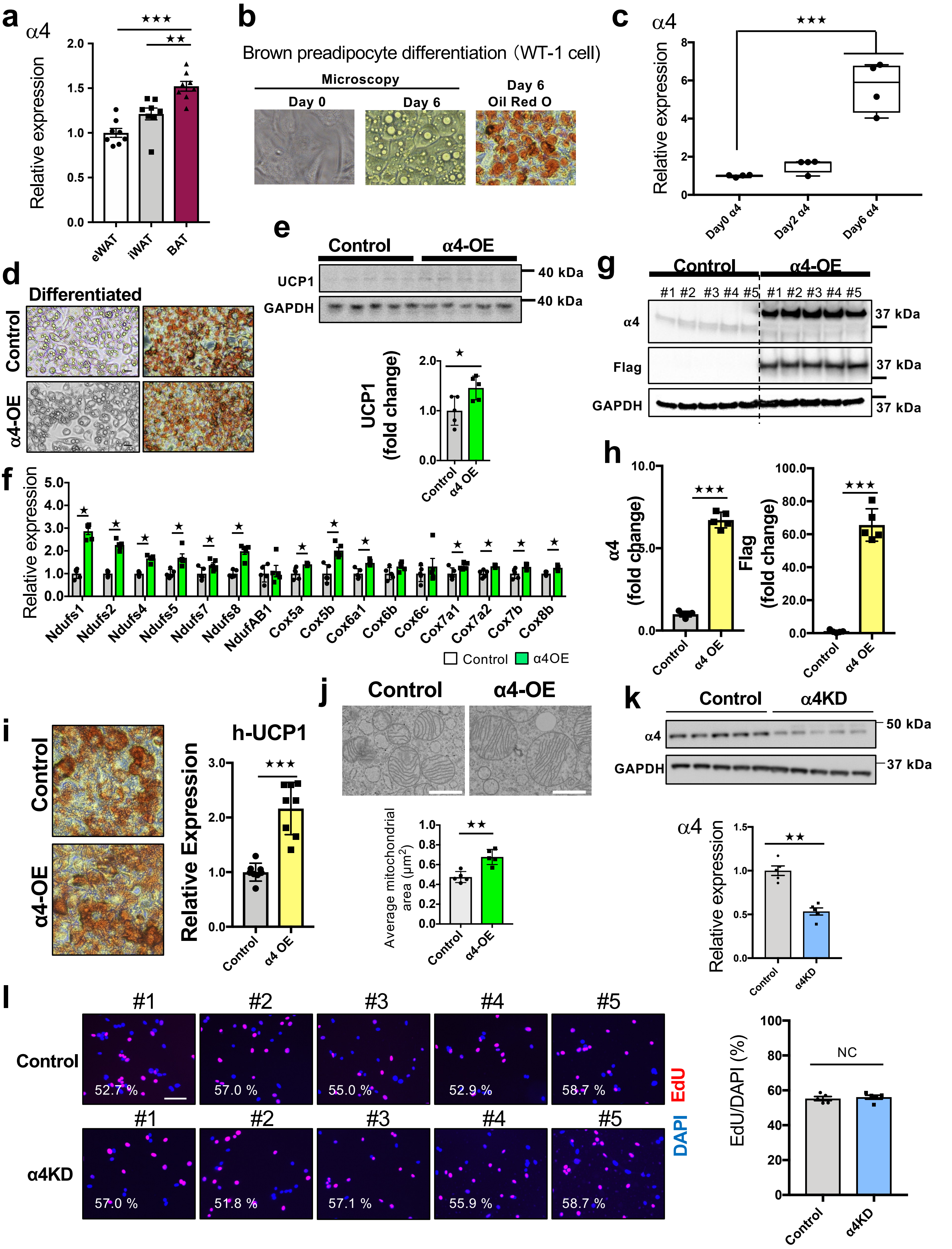

a
α4
Relative expression
eWAT
iWAT
BAT
b
Brown preadipocyte differentiation (WT-1 cell)
Microscopy
Day 0
Day 6
Day 6 Oil Red O
c
α4
Relative expression
Day0 α4
Day2 α4
Day6 α4
d
Differentiated
Control
α4-OE
e
Control
α4-OE
UCP1
GAPDH
40 kDa
40 kDa
UCP1 (fold change)
Control
α4 OE
f
Relative expression
Ndufs1
Ndufs2
Ndufs4
Ndufs5
Ndufs7
Ndufs8
NdufAB1
Cox5a
Cox5b
Cox6a1
Cox6b
Cox6c
Cox7a1
Cox7a2
Cox7b
Cox8b
Control
α4OE
g
Control
α4-OE
#1 #2 #3 #4 #5 #1 #2 #3 #4 #5
α4
Flag
GAPDH
37 kDa
37 kDa
37 kDa
h
α4 (fold change)
Flag (fold change)
Control
α4 OE
i
Control
α4-OE
h-UCP1
Relative Expression
Control
α4 OE
j
Control
α4-OE
Average mitochondrial area (μm²)
Control
α4-OE
k
Control
α4KD
α4
GAPDH
50 kDa
37 kDa
α4
Relative expression
Control
α4KD
l
#1 #2 #3 #4 #5
Control
52.7 %
57.0 %
55.0 %
52.9 %
58.7 %
α4KD
57.0 %
51.8 %
57.1 %
55.9 %
58.7 %
EdU
DAPI
EdU/DAPI (%)
NC
Control
α4KD

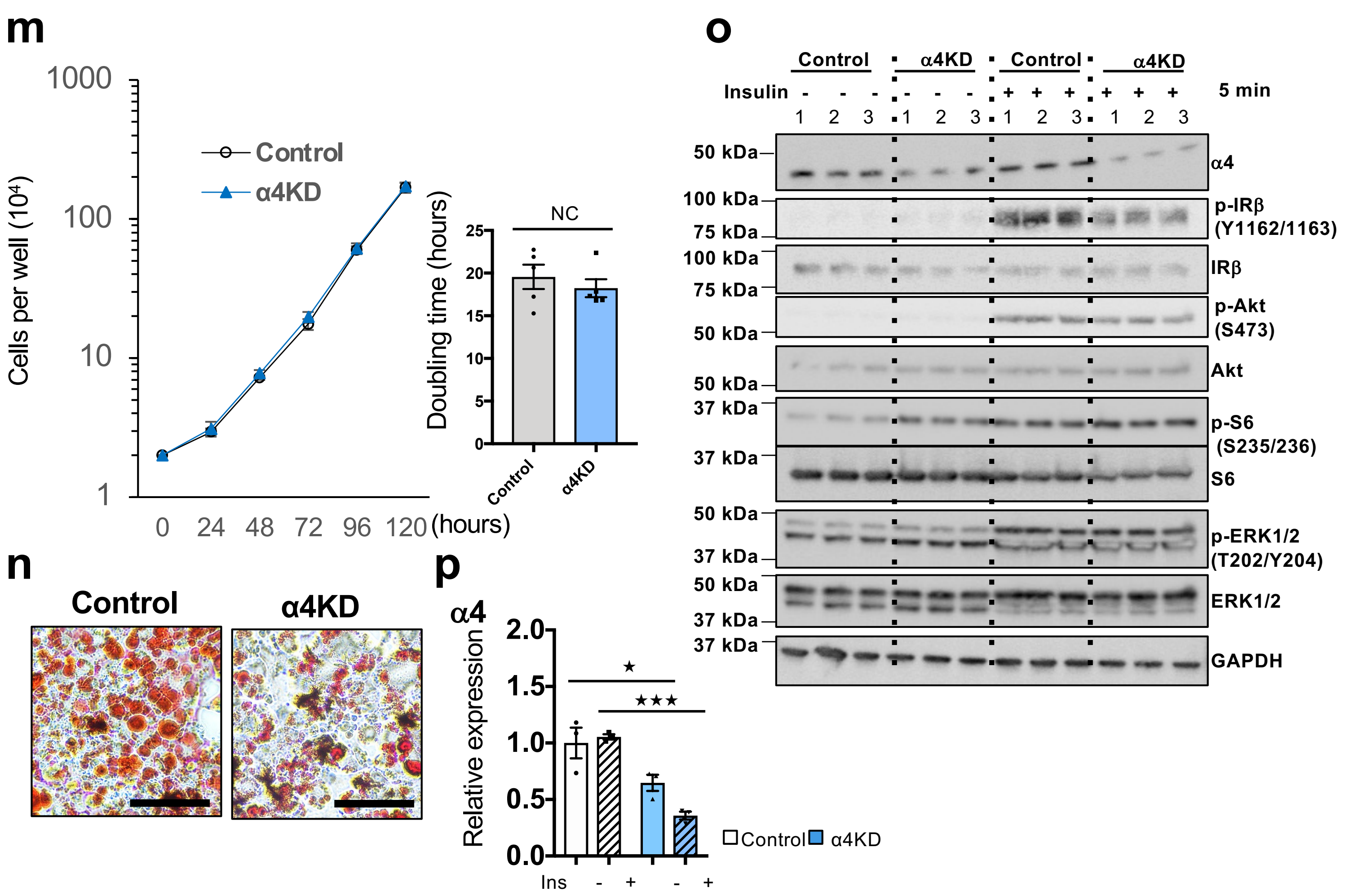
m
Cells per well (10^4)
1000
100
10
1
Control
α4KD
0
24
48
72
96
120
(hours)
Doubling time (hours)
NC
25
20
15
10
5
0
Control
α4KD
n
Control
α4KD
o
Control
α4KD
Control
α4KD
Insulin
5 min
1 2 3 1 2 3 1 2 3 1 2 3
50 kDa
100 kDa
75 kDa
100 kDa
75 kDa
50 kDa
50 kDa
37 kDa
37 kDa
50 kDa
37 kDa
50 kDa
37 kDa
37 kDa
α4
p-IRβ (Y1162/1163)
IRβ
p-Akt (S473)
Akt
p-S6 (S235/236)
S6
p-ERK1/2 (T202/Y204)
ERK1/2
GAPDH
p
α4
Relative expression
2.0
1.5
1.0
0.5
0.0
Ins
Control
α4KD

Supplementary Figure 1. Knockdown of α4 in brown preadipocytes decreased insulin signaling.
(**a**) α4 expression (qPCR) from three different adipose tissues of 5-month-old C57BL/6J mice. Data are presented as mean ± SEM (One-way ANOVA post hoc Bonferroni test, ** p = 0.003, *** p < 0.0001, n = 8). (**b**) The bright-field microscopy image of the differentiated brown preadipocytes corresponds to Days 0 and 6; Scale bar = 100 µm. The results of Oil red O staining corresponding to Day 6 are also shown. **(c)** Densitometric analysis of α4 in lysates from brown preadipocyte cells during the differentiation process before or 2 and 6 days after induction of the differentiation. Box plots are defined in terms of minima and maxima by whiskers, and the center and bounds of box by quartiles (One-way ANOVA post hoc Bonferroni test, n = 4, *** p < 0.0001) **(d)** Bright field microscopy picture (left) and Oil red O staining (right) of the mouse differentiated brown adipocyte from Control and overexpressed 3XFlag-α4 (α4 OE) cells. The experiments were repeated independently three times. Scale bars, 100 µm. **(e)** Immunoblotting and densitometric analysis of UCP1 in lysates from differentiated adipocytes of α4-OE cells. Data are mean ± SEM (two-tailed Student t-test, * p = 0.02, n = 5 per group). **(f)** Mitochondrial markers in differentiated brown adipocytes from Control and 3XFlag-α4 transfected (α4-OE) mice cells. Data are mean ± SEM (two-tailed Student t-test, n = 5 per group, * p < 0.05). **(g)** Immunoblotting for α4 and Flag in lysates from Control human brown preadipocytes or cells overexpressed 3XFlag-α4 (α4-OE). (n = 5 biologically independent cell clones/group) **(h)** Densitometric analysis of α4 and Flag in human brown preadipocytes. Data are mean ± SEM of n = 5 (two-tailed Student t-test, *** p < 0.0001). (**i**) Representative Oil red O staining (left) and *UCP1* mRNA expression (right) in human differentiated brown adipocytes from Control (n = 8) and 3XFlag-α4-OE cells (n = 8). Data are mean ± SEM (two-tailed Student t-test, *** p < 0.0001).(**j**) (upper) Representative electron microscopic images of mitochondria in differentiated brown adipocytes from Control and α4-OE cells. Scale bar, 1.0 µm. (bottom) Quantification of the average mitochondrial size from Control and α4OE brown adipocytes. Data are mean ± SEM (Two-tailed Student's t-test, ** p = 0.001; n = 5). **(k)** Immunoblotting and densitometric analysis of α4 in lysates from preadipocytes depleted of α4 (α4 KD) by shRNA. Data are mean ± SEM (two-tailed Student t-test, ** p = 0.0001; five clones used in the experiments). **(l)** Representative images of EdU (Red) incorporation in Control and α4KD cells. Scale bars, 100 µm (left). Quantification of BrdU+ DAPI+ cells (right). Data are mean ± SEM (two-tailed Student t-test, five clones with the quadruplicate were used). **(m)** Cells were plated at a density of 20,000 cells per well and their number determined at 24h intervals for 5 consecutive days (left). Doubling time was calculated during the exponential growth phase between day 3 and day 4 after plating (right). Data are mean ± SEM (five clones were used in the experiments).**(n)** Oil red O staining of the mouse differentiated brown adipocyte from Control and α4 KD cells. The experiments were repeated independently three times. Scale bars, 80 µm. **(o)** Western blot analysis of insulin signaling in lysates from α4 KD preadipocytes 5 min after 100 nM insulin stimulation for indicated antibodies. Three clones used in three independent experiments. (**p**) Relative changes of a4 based on the densitometric immunoblotting analysis (**Supplementary Figure 1i**) of cell lysates of adipocytes from α4KD-treated and Control mice for 5 min using 100 nM insulin. Data are presented as mean ± SEM (One-way ANOVA post hoc Bonferroni test, * p = 0.02, *** p = 0.0005, Three clones used in three independent experiments.).

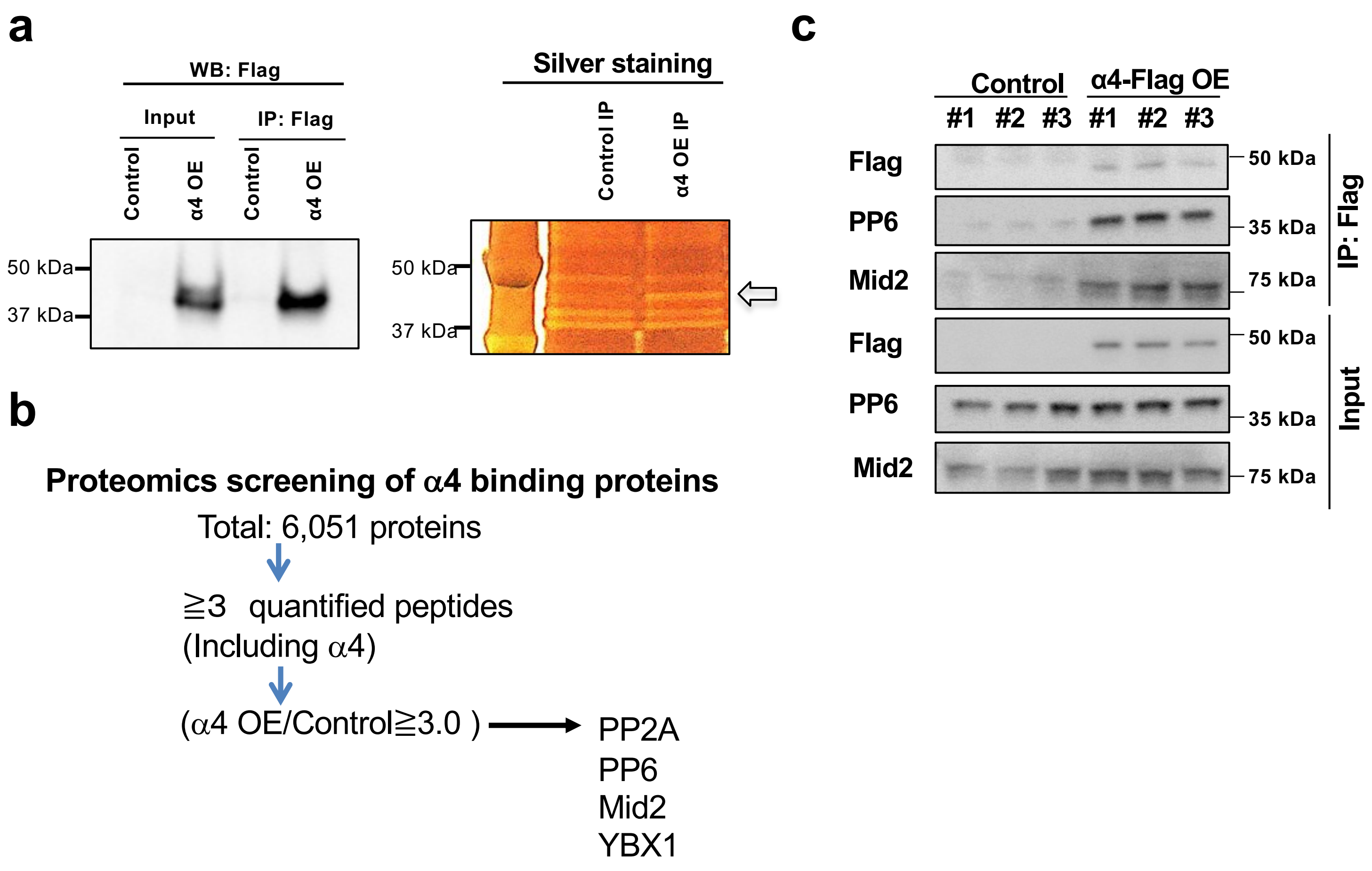

Supplementary Figure 2. Identification of α4 binding protein which regulates IR tyrosine phosphorylation.

(a) Flag-tagged α4 associated with the protein complex immunoprecipitated using Flag beads from the lysates of brown adipocytes as in (**Fig. 2a**) and subjected to SDS-PAGE with silver staining.

(b) Proteomics screening of α4 binding proteins. For the mass spectroscopic proteomic analysis. we compared the Control (n = 1) and α4 overexpressing (n = 2, biological replicates) samples.

(c) Flag-tagged α4 immunoprecipitation showed the interaction between Mid2 with α4 as well as that between PP6 and α4 in brown preadipocytes.

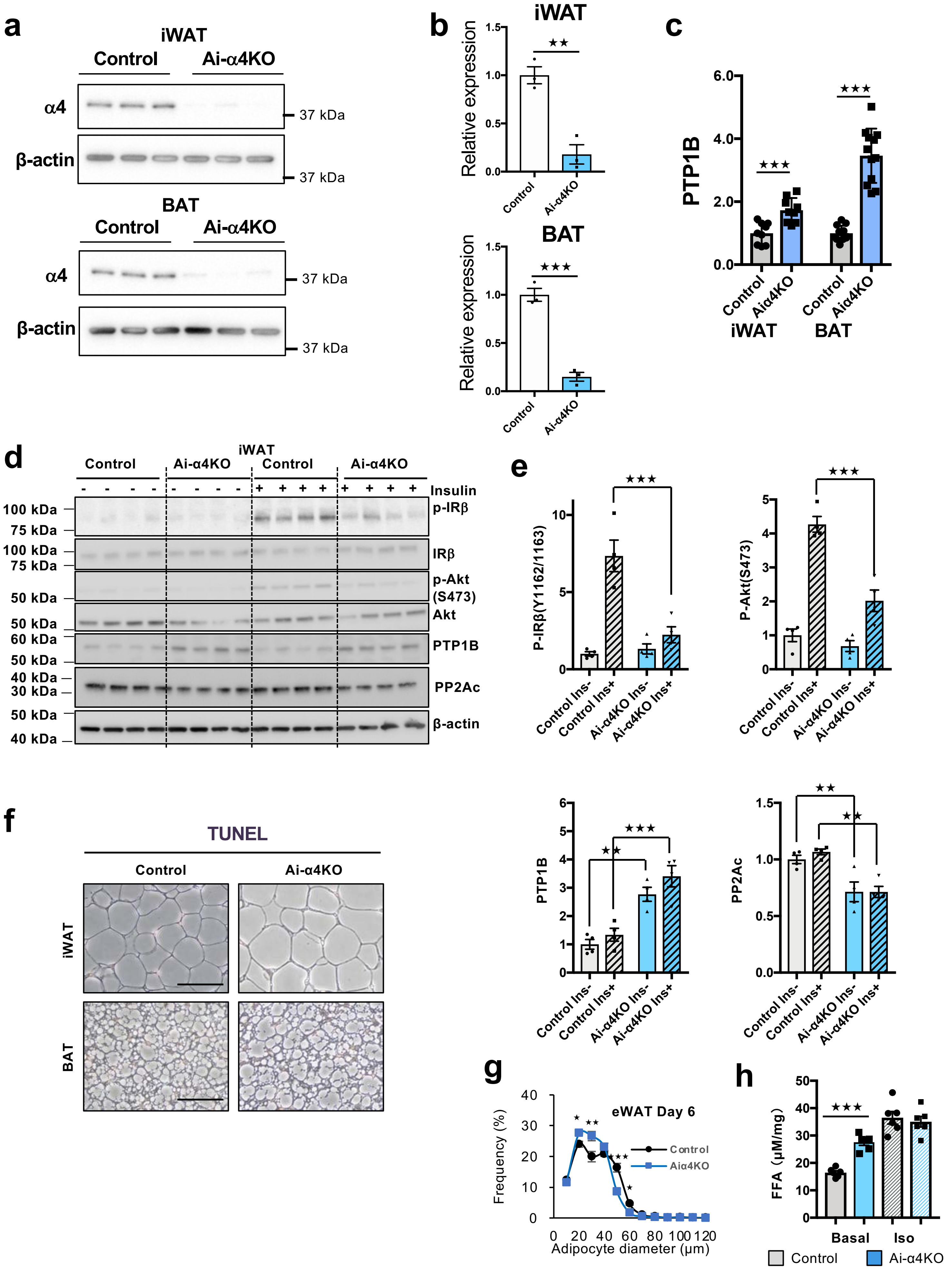
a
iWAT
Control
Ai-α4KO
α4
β-actin
37 kDa
37 kDa
BAT
Control
Ai-α4KO
α4
β-actin
37 kDa
37 kDa
b
iWAT
Relative expression
Control
Ai-α4KO
BAT
Relative expression
Control
Ai-α4KO
c
PTP1B
Control
Aiα4KO
Control
Aiα4KO
iWAT
BAT
d
iWAT
Control
Ai-α4KO
Control
Ai-α4KO
Insulin
100 kDa
75 kDa
p-IRβ
100 kDa
75 kDa
IRβ
p-Akt
(S473)
50 kDa
50 kDa
Akt
60 kDa
50 kDa
PTP1B
40 kDa
30 kDa
PP2Ac
50 kDa
40 kDa
β-actin
e
P-IRβ(Y1162/1163)
P-Akt(S473)
PTP1B
PP2Ac
Control Ins-
Control Ins+
Ai-α4KO Ins-
Ai-α4KO Ins+
f
TUNEL
Control
Ai-α4KO
iWAT
BAT
g
eWAT Day 6
Frequency (%)
Control
Aiα4KO
Adipocyte diameter (μm)
h
FFA (μM/mg)
Basal
Iso
Control
Ai-α4KO

i

Abbreviation	Name	Group
CE	Cholesteryl ester	Neutral Lipids
MAG	Monoacylglycerol	
DAG	Diacylglycerol	
TAG	Triacylglycerol	
PC	Phosphatidylcholine	Phospholipids
PE	Phosphatidylethanolamine	
PI	Phosphatidylinositol	
LPC	Lysophosphatidylcholine	
LPE	Lysophosphatidylethanolamine	
SM	Sphingomyelin	Sphingolipids
CER	Ceramide	
HCER	Hexosylceramide	
LCER	Lactosylceramide	
DCER	Dihydroceramide	

j

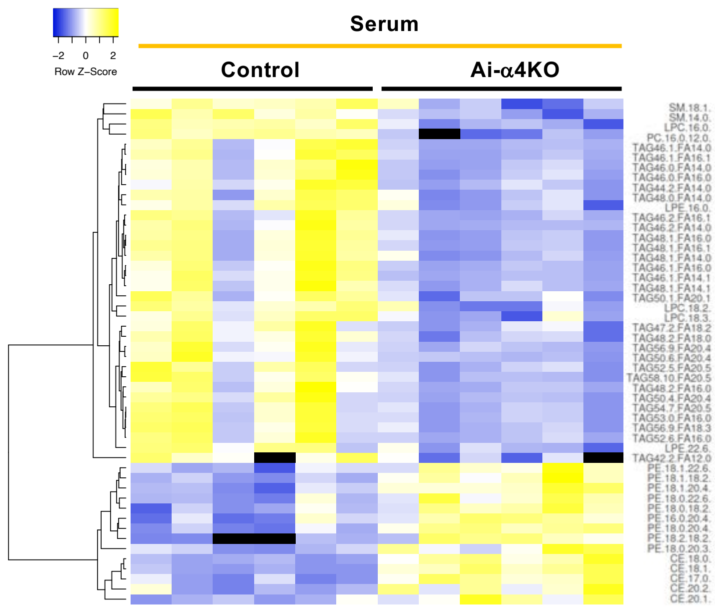

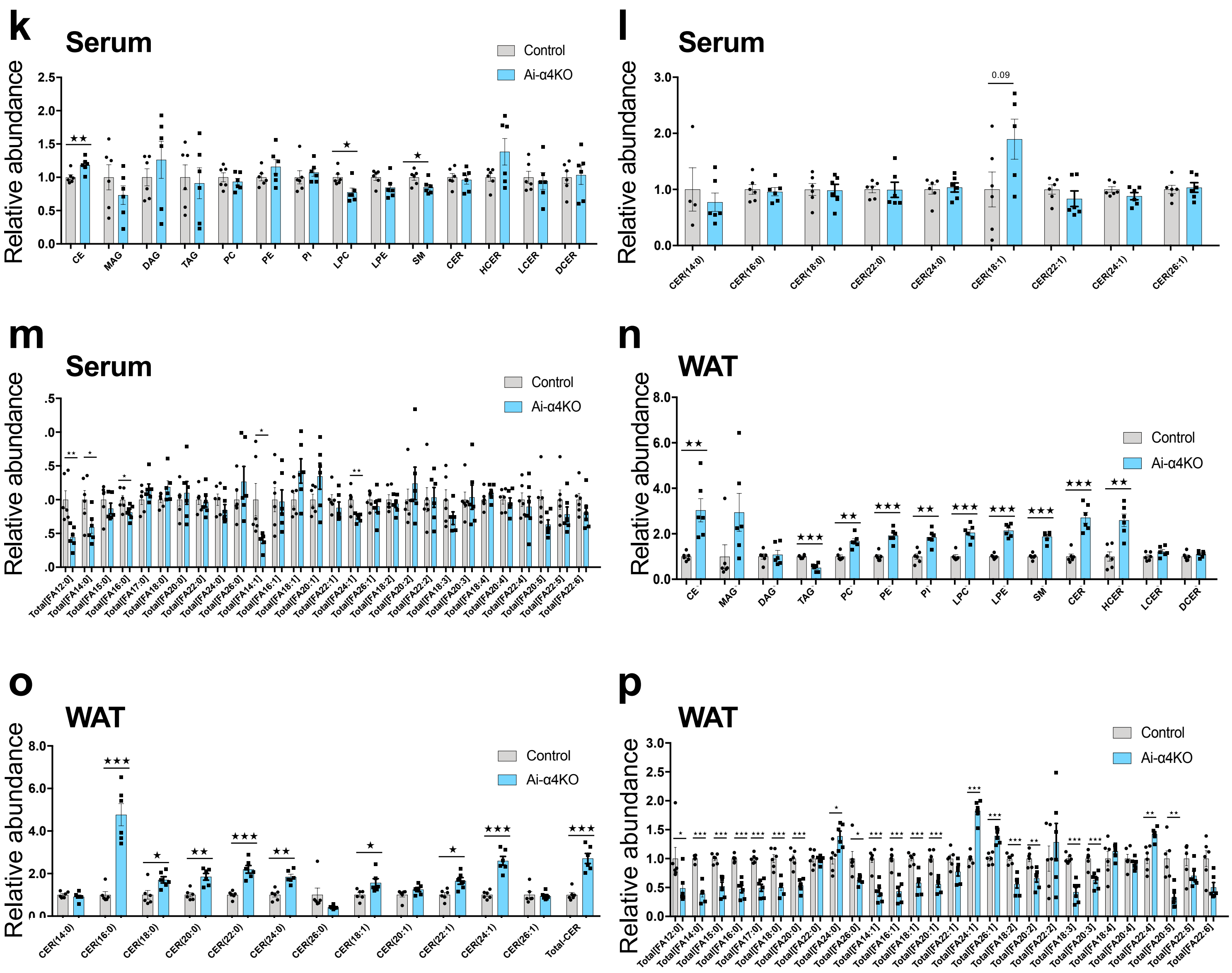

Supplementary Figure 3. Acute loss of α4 in adipocytes altered serum lipid profiles.
(a) Western blot analysis and **(b)** densitometric analysis of α4 in inguinal WAT **(upper)** and interscapular BAT **(bottom)** of Control and Ai-α4KO mice at day 3. Data are mean ± SEM (two-tailed Student t-test, ** p = 0.003, *** p = 0.0004, n = 3). (**c**) YBX1 mRNA expression measured by real-time qPCR in iWAT (n = 9 per genotype) and BAT (n = 12 per genotype) from Control and Ai-α4KO. Data are mean ± SEM (two-tailed Student t-test, iWAT: *** p = 0.0006, BAT: *** p < 0.0001). Western blot analysis of insulin signaling **(d)** and densitometric analysis of phosphorylated IR, phosphorylated Akt and PTP1B in inguinal WAT **(e)** of 3-months-old fasted Control and Ai-α4KO mice 10 min after i.v. insulin stimulation (5 IU per mouse) or control saline (n = 4). Data are mean ± SEM (One-way ANOVA post hoc Bonferroni test: * p < 0.05; ** p < 0.01; *** p < 0.001). (**f**) TUNEL staining results corresponding to iWAT and BAT sections from Control and Ai-α4KO mice on Day 3. Scale bars =50 μm. **(g)** Diameter distribution of isolated eWAT adipocytes in Control and and Ai-α4KO at day 6. Data are mean ± SEM (two-tailed Student t-test, *, p < 0.05; **, p < 0.01; *** p < 0.001, n = 8/group). (**h**) Lipolysis assessed by FFA release from eWAT of Control and and Ai-α4KO mice on Day 6. Samples were incubated ex vivo in the presence or absence of 10 mM isoprenaline, and FFA release into the medium was quantified. (Two-tailed Student's t-test, *** p = 0.0006: n = 6/group). **(i)** Quantified lipid classes and their abbreviations. **(j)** Heatmap of top 50 lipid species from serum differentially regulated between Control and Ai-α4KO in mice (n = 5 per group) at 1week. Lipid class concentrations of Serum **(k)** and iWAT **(n)** from Ai-α4KO expressed as 1.0 of Control. Fatty acid concentrations of the indicated chain lengths were quantified in Serum **(l)** and iWAT **(o)** from Ai-α4KO expressed as 1.0 of Control. Ceramide species of the indicated chain lengths were quantified in Serum **(m)** and iWAT **(p)** from Ai-α4KO expressed as 1.0 of Control. Data are mean ± SEM (two-tailed Student t-test, * p < 0.05; ** p < 0.01; *** p < 0.001, n = 5 per group).

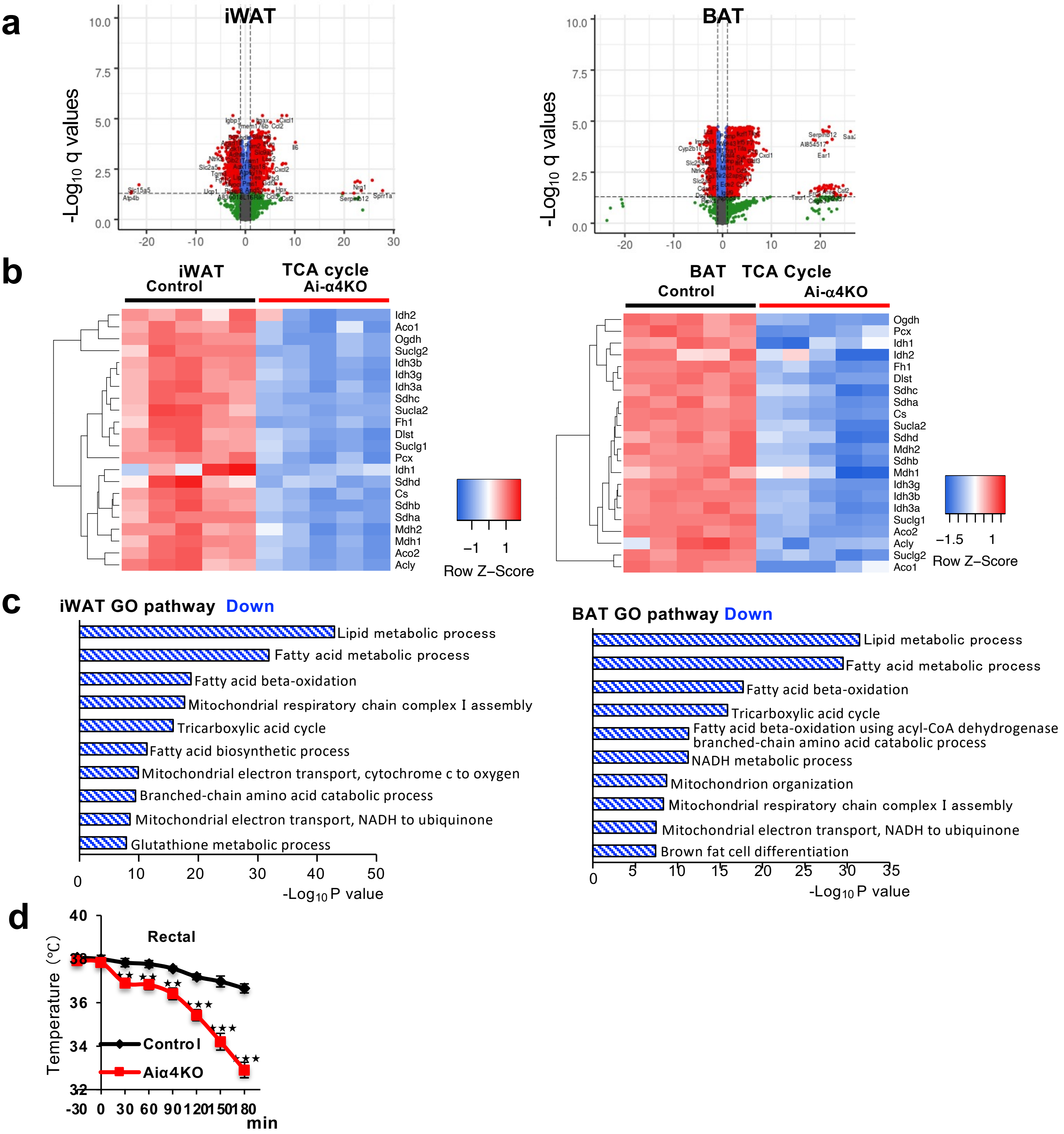

Supplementary Figure 4. Acute loss of α4 reduces a Mitochondrial Biogenesis gene signature in adipose tissues.

(a) Volcano plot showing the distribution of differentially regulated genes in iWAT (left) and BAT (right), with log ratio of fold change in Control versus fold change in Ai-α4KO on x axis and -log10 q value on y axis. **(b)** Genes involved in the TCA cycle pathway in iWAT and BAT were listed in the heatmap, and the color intensities indicate Z-score of each sample by gene.

(c) Top ten directionally down-regulated GO pathways between Control and Ai-α4KO in iWAT (left) and BAT (right). **(d)** Rectal temperature of Control (n = 6) and Ai-α4KO (n = 5) mice on Day 10 during a 3-h exposure to an environment at 4 ° C. Data are mean ± SEM (Two-tailed Student's t-test: * $p < 0.05$; ** $p < 0.01$; *** $p < 0.001$).

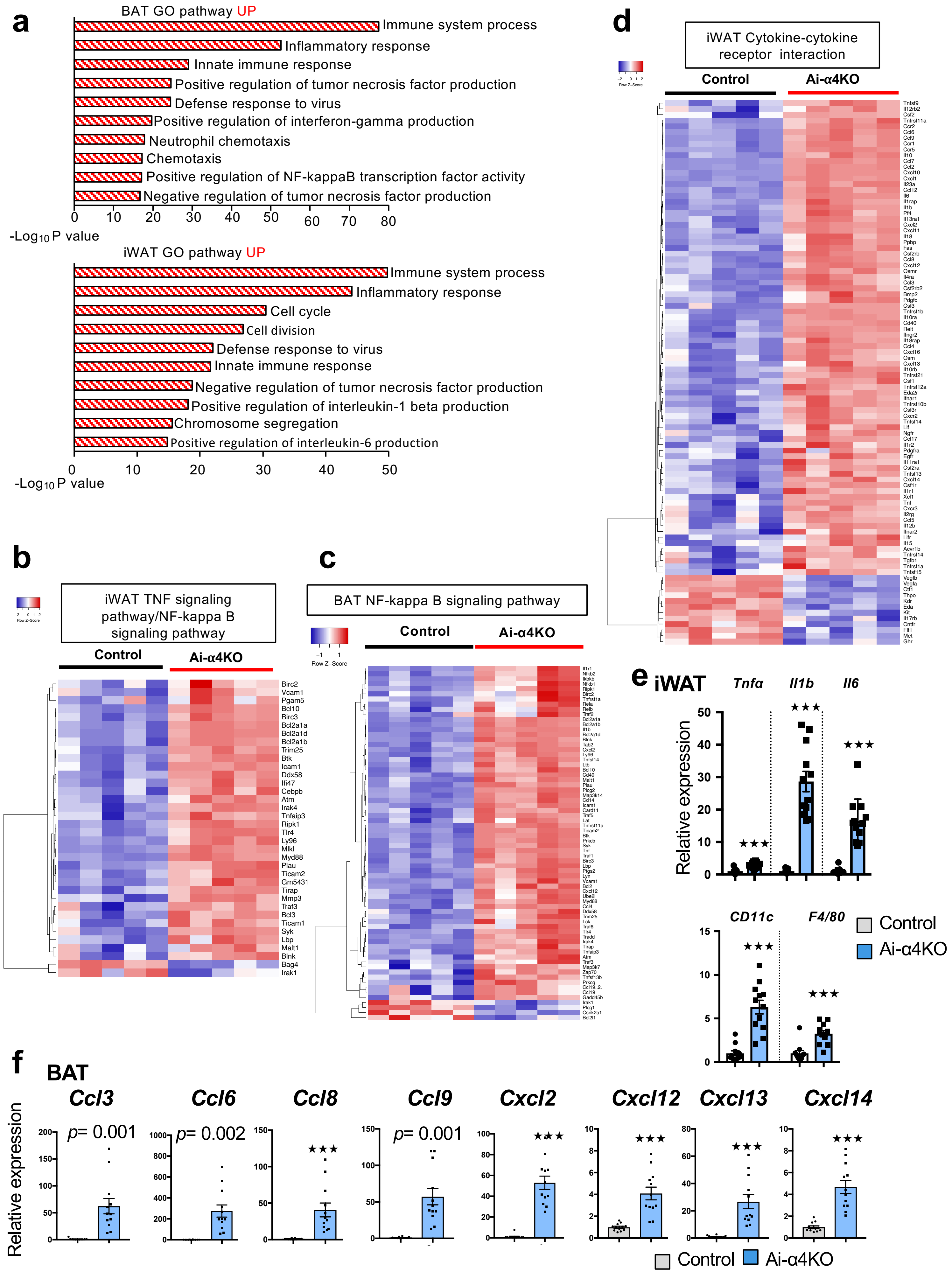
a
BAT GO pathway UP
Immune system process
Inflammatory response
Innate immune response
Positive regulation of tumor necrosis factor production
Defense response to virus
Positive regulation of interferon-gamma production
Neutrophil chemotaxis
Chemotaxis
Positive regulation of NF-kappaB transcription factor activity
Negative regulation of tumor necrosis factor production
0 10 20 30 40 50 60 70 80
-Log10 P value
iWAT GO pathway UP
Immune system process
Inflammatory response
Cell cycle
Cell division
Defense response to virus
Innate immune response
Negative regulation of tumor necrosis factor production
Positive regulation of interleukin-1 beta production
Chromosome segregation
Positive regulation of interleukin-6 production
0 10 20 30 40 50
-Log10 P value
b
iWAT TNF signaling pathway/NF-kappa B signaling pathway
Control
Ai-α4KO
Birc2 Vcam1 Pgam5 Bcl10 Birc3 Bcl2a1a Bcl2a1d Bcl2a1b Trim25 Btk Icam1 Ddx58 Ifi47 Cebpb Atm Irak4 Tnfaip3 Ripk1 Tlr4 Ly96 Mlkl Myd88 Plau Ticam2 Gm5431 Tirap Mmp3 Traf3 Bcl3 Ticam1 Syk Lbp Malt1 Blnk Bag4 Irak1
c
BAT NF-kappa B signaling pathway
Row Z-Score
Control
Ai-α4KO
d
iWAT Cytokine-cytokine receptor interaction
Control
Ai-α4KO
e
iWAT
Tnfa Il1b Il6
Relative expression
0 10 20 30 40 50
CD11c F4/80
0 5 10 15
Control
Ai-α4KO
f
BAT
Ccl3 Ccl6 Ccl8 Ccl9 Cxcl2 Cxcl12 Cxcl13 Cxcl14
p= 0.001
p= 0.002
p= 0.001
Relative expression
Control
Ai-α4KO

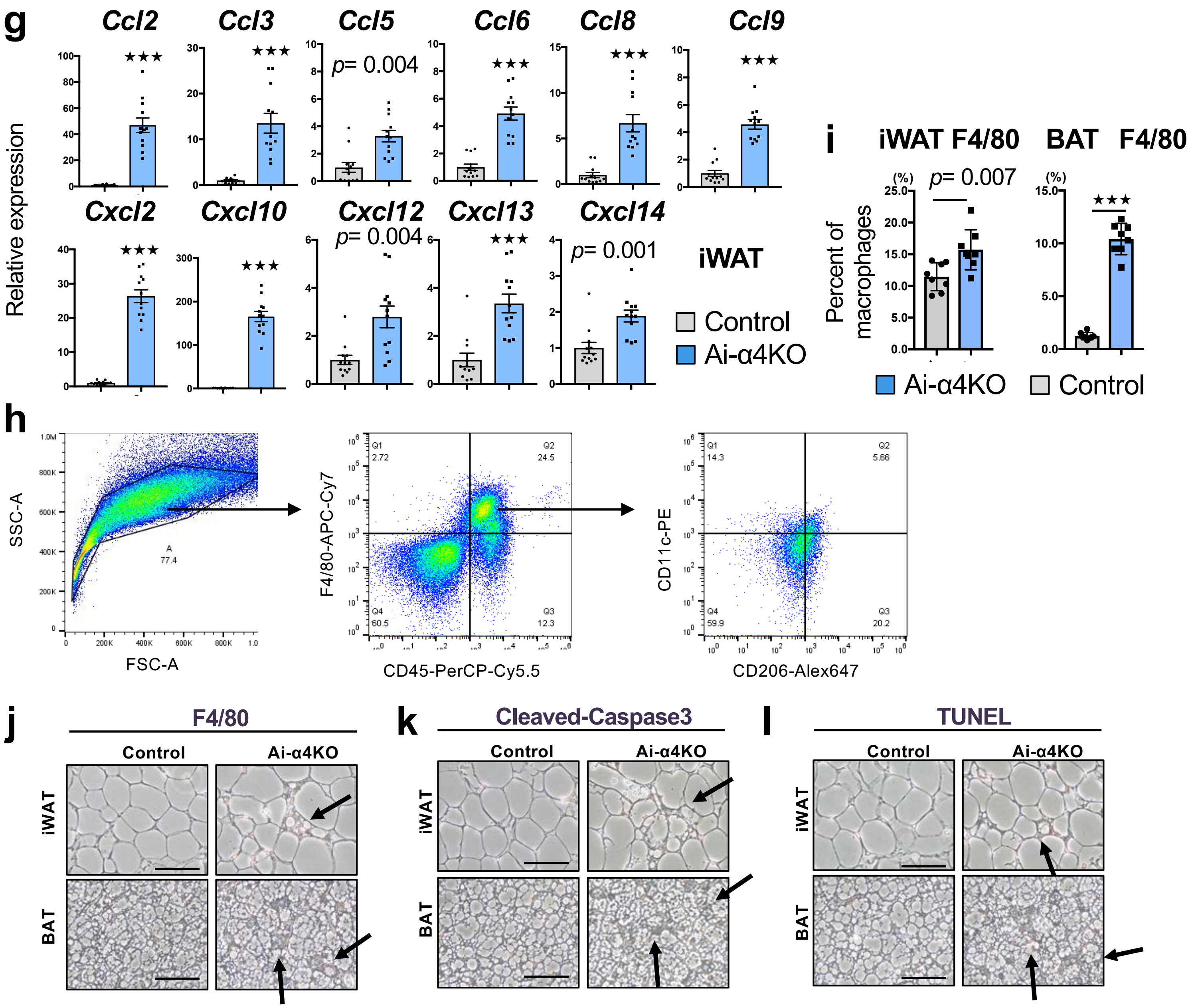

Supplementary Figure 5. Acute loss of α4 induces inflammatory chemokines and chemokine receptors gene signatures in adipose tissues.
(a) Top directionally up-regulated GO pathways between Control and Ai-α4KO in BAT (upper) and iWAT (bottom). Heatmap of Genes involved in the cytokine-cytokine receptor interaction **(b)** in iWAT and TNF/NF-kappa B signaling pathways in iWAT **(c)** and BAT **(d)** were listed in the heatmap, and the color intensities indicate Z-score of each sample by gene. **(e)** Expression levels of inflammatory cytokines and inflammasome components measured by real-time qPCR in iWAT from Control and Ai-α4KO at day 10 (n = 11 per group). Expression levels of genes for chemokines were measured by real-time qPCR in BAT (**f**) and iWAT (**g**) from Control and Ai-α4KO (n = 12 per group). **(h)** Flow cytometry gating strategy of macrophage populations in the adipose tissues. Representative dot plots showing Single cells, Live cells and C + cells were sorted for CD45+ and F4/80+ and were then sorted on the basis of expression of CD11c and CD206. (**i**) Percent of macrophages (F4/80+) infiltrated in iWAT and BAT in Control and Ai-α4KO mice by flowcytometric analysis (n = 8 per group). Staining of iWAT and BAT sections from Control and Ai-α4KO mice on Day 6 for (**j**) F4/80 and (**k**) cleaved-caspase3. (**l**) TUNEL staining results corresponding to iWAT and BAT sections from Control and Ai-α4KO mice on Day 6. Scale bars =50 μm. The red arrow shows TUNEL positive cells. Experiments in j–l were repeated three times independently. All data are mean ± SEM (two-tailed Student t-test). Statistical significance is shown as p <0.05 (the exact p value) *or* p <0.001 (***).

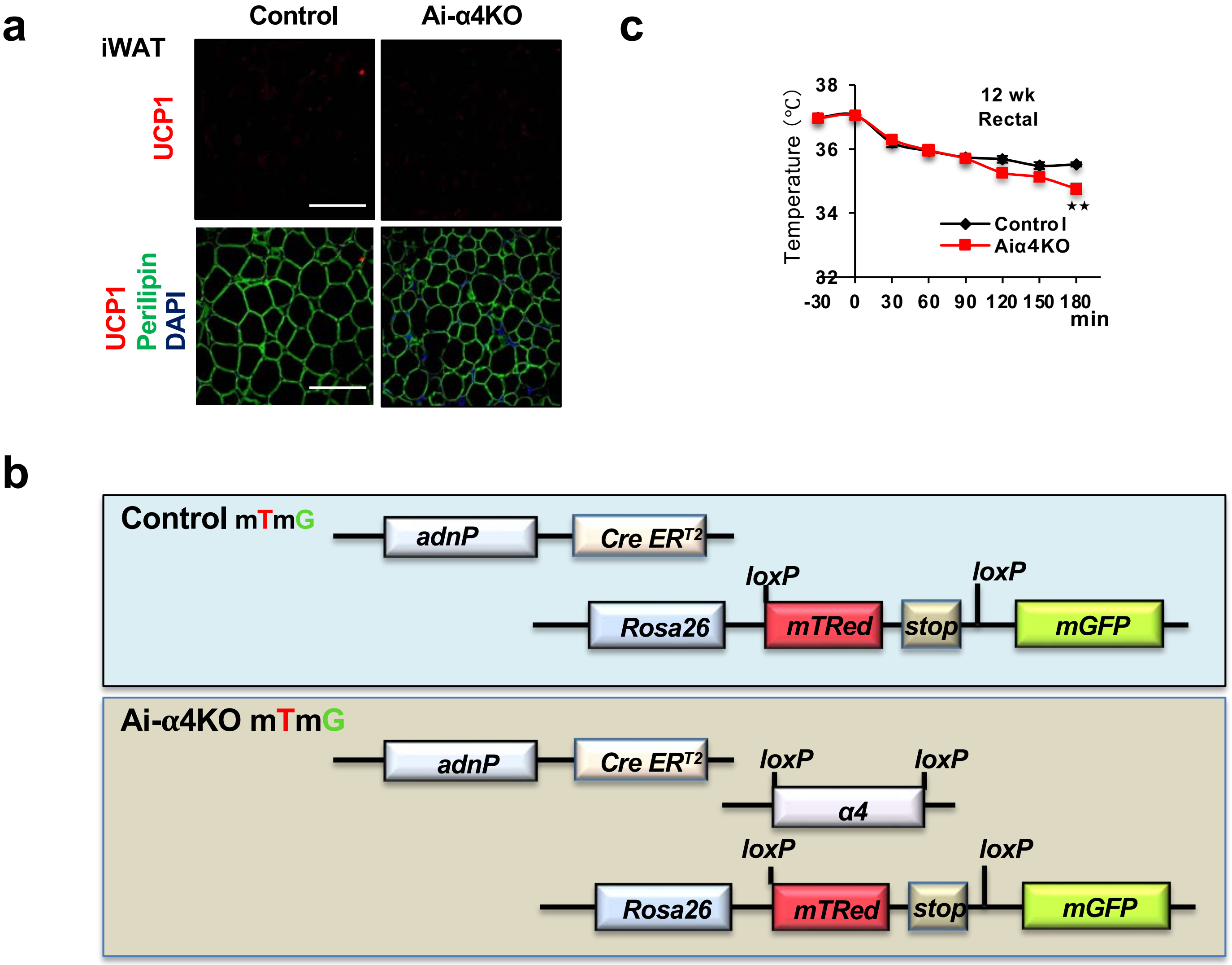

Supplementary Figure 6. Adipose tissue biology in Control and Ai-α4KO mice.
(a) UCP1 expression in iWAT from Control and Ai-α4KO mice on Days 74. Scale bars = 100 μm. **(b)** Strategy for identifying newly developed adipocytes in Ai-α4KO mice in combination with floxed *mTmG* mouse. **(c)** Rectal temperature in male Control and Ai-α4KO mice at 30-min intervals during the 3-h exposure of mice aged 12 wks to an environment at 4 ° C. Data are mean ± SEM (Two-tailed Student's t-test: ** *p* = 0.002; n = 5)

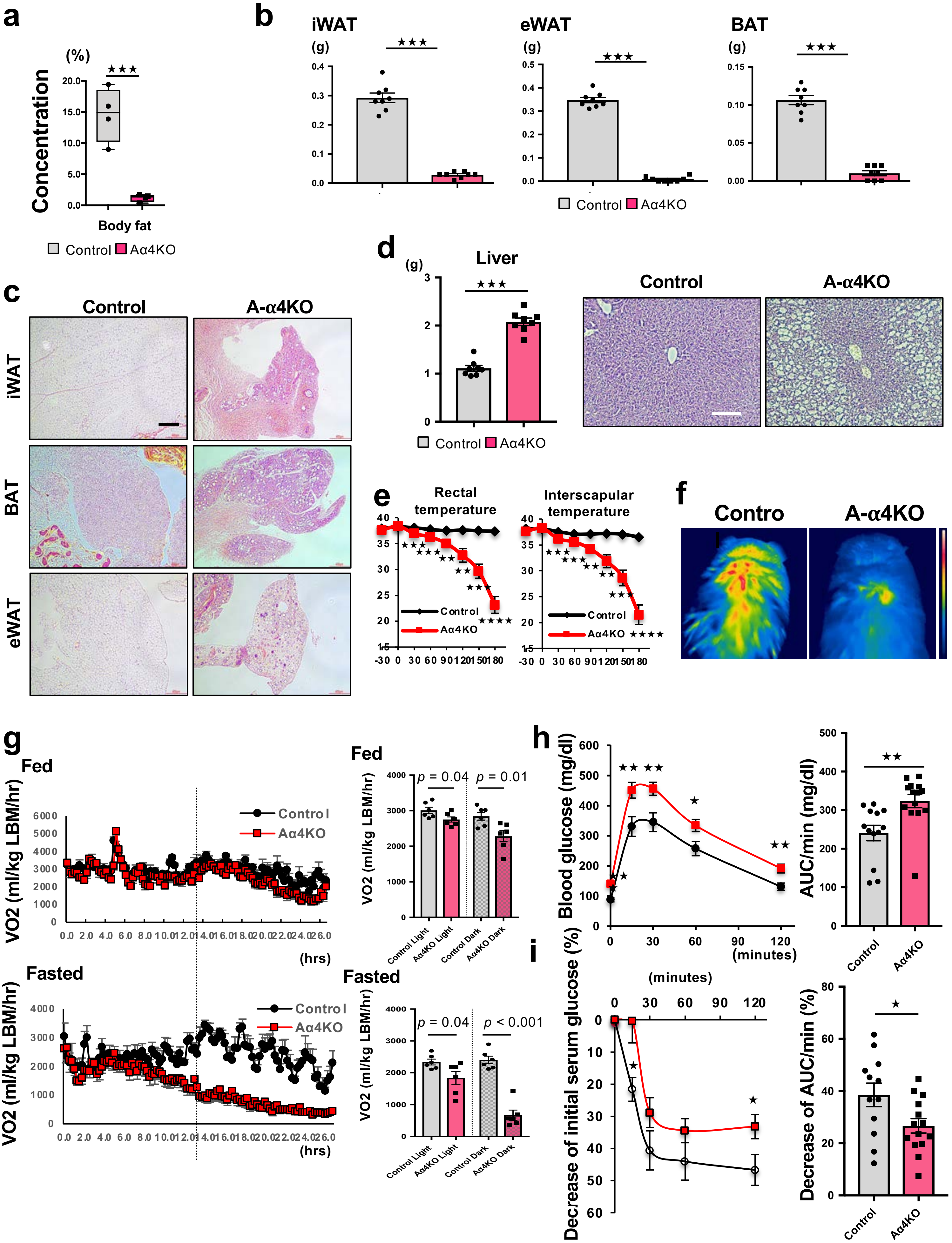

a
Concentration (%)
Body fat
Control Aα4KO
b
iWAT
eWAT
BAT
(g)
Control Aα4KO
c
Control
A-α4KO
iWAT
BAT
eWAT
d
Liver
(g)
Control Aα4KO
Control
A-α4KO
e
Rectal temperature
Interscapular temperature
Control
Aα4KO
f
Contro
A-α4KO
g
Fed
VO2 (ml/kg LBM/hr)
Control
Aα4KO
(hrs)
Fed
p = 0.04
p = 0.01
Control Light
Aα4KO Light
Control Dark
Aα4KO Dark
Fasted
Control
Aα4KO
(hrs)
Fasted
p = 0.04
p < 0.001
h
Blood glucose (mg/dl)
(minutes)
AUC/min (mg/dl)
Control
Aα4KO
i
Decrease of initial serum glucose (%)
(minutes)
Decrease of AUC/min (%)
Control
Aα4KO

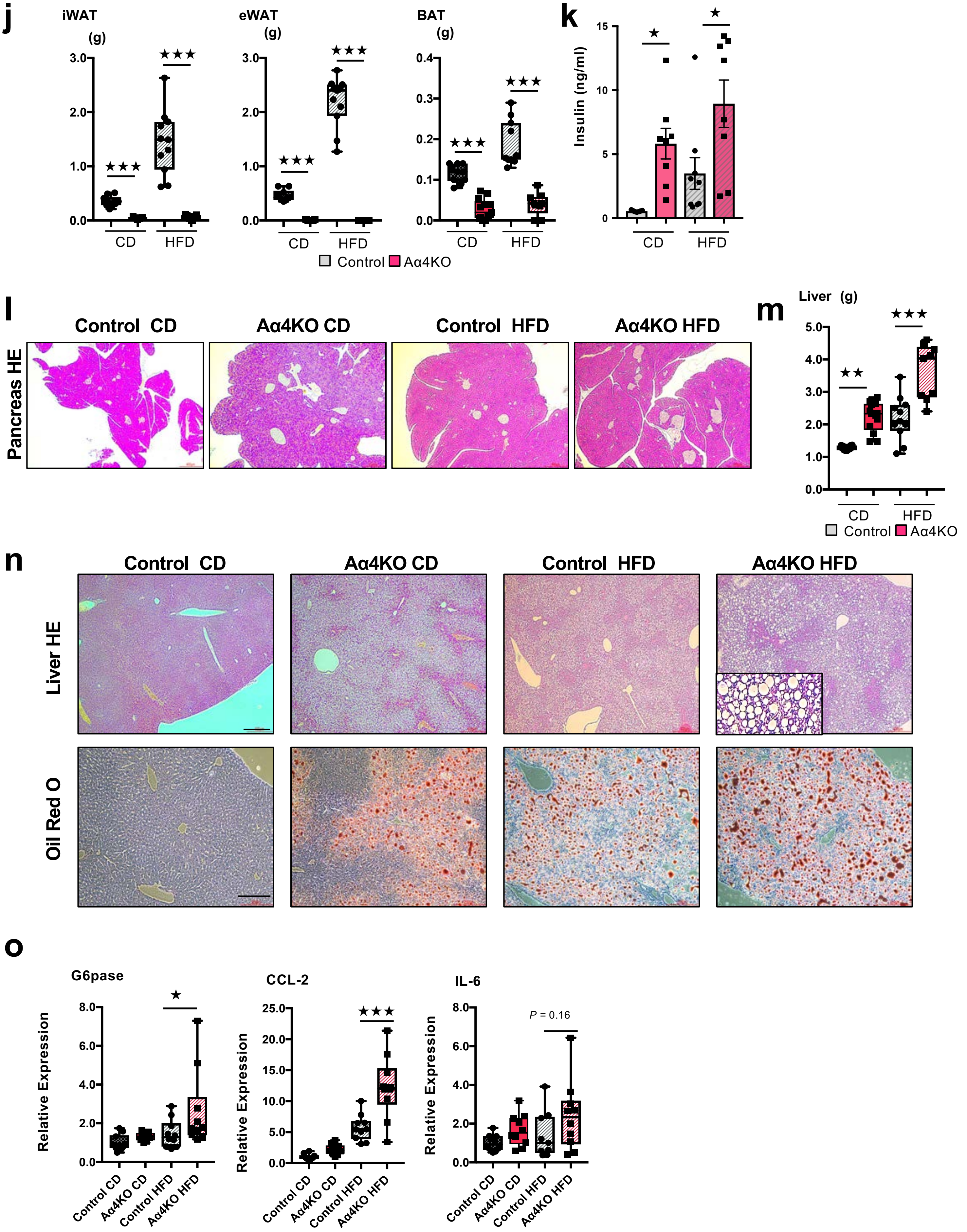

j
iWAT
eWAT
BAT
(g)
CD
HFD
k
Insulin (ng/ml)
Control
Aα4KO
l
Control CD
Aα4KO CD
Control HFD
Aα4KO HFD
Pancreas HE
m
Liver (g)
n
Liver HE
Oil Red O
o
G6pase
CCL-2
IL-6
Relative Expression
P = 0.16

Supplementary Figure 7. Aα4KO mice showed cold intolerance, diabetes, pancreatic islet hyperplasia and fatty liver .

(**a**) Body fat percentage calculated by micro-CT imaging of Control (n = 4) and Aα4KO (n = 4). Box plots are defined in terms of minima and maxima by whiskers, and the center and bounds of box by quartiles (two-tailed Student t-test, *** p = 0.0009). (**b**) Tissue weights of iWAT, eWAT and BAT in 3 months old Control (n = 8) and Aα4KO (n = 8). Data are mean ± SEM (two-tailed Student t-test, *** p < 0.0001). (**c**) HE stained sections of iWAT, eWAT and BAT in Control and Aα4KO. Scale bars, 400 µm. (**d**) Tissue weights of Liver in 3 months old Control (n = 8) and Aα4KO (n = 8). Data are mean ± SEM (two-tailed Student t-test, *** p < 0.0001) (left). HE stained sections of Liver in Control and Aα4KO. Scale bars, 100 µm (right). (**e**) Rectal and interscapular temperature in Control and Aα4KO during a 3 h exposure to 4℃ environment. Data are mean ± SEM (two-tailed Student t-test, *, p < 0.05; **, p < 0.01; *** p < 0.001; **** p < 0.0001, n = 5 per group). (**f**) Thermal images using a FLIR E8 Infrared Camera showing surface temperature over interscapular BAT after 2 h at 4 ℃ between Control and Aα4KO. (**g**) Oxygen consumption (VO2) measured in metabolic cages with Control (n = 6) and Aα4KO (n = 6) mice under fed (**upper**) and fasted (**bottom**) condition. Dark phase is the 12h period of day during which the lights were off. (two-tailed Student t-test). All data are represented as mean ± SEM. (**h**) GTT and GTT AUC for Control (n = 13) and Aα4KO (n = 14). Data are mean ± SEM (two-tailed Student t-test, ** p = 0.004). (**i**) ITT and the decrease of AUC in Control (n = 12) and Aα4KO (n = 14). Data are mean ± SEM (two-tailed Student t-test, * p = 0.02). (**j**) Tissue weights of iWAT, eWAT and BAT in Control and Aα4KO mice fed a chow diet or HFD. Box plots are defined in terms of minima and maxima by whiskers, and the center and bounds of box by quartiles (One-way ANOVA post hoc Bonferroni test, *** p < 0.001, Control CD (n = 10), Aα4KO CD (n = 13), Control HFD (n = 11) and Aα4KO HFD (n = 9)). (**k**) Serum insulin levels of Control and Aα4KO mice fed a chow diet or HFD. Data are mean ± SEM (One-way ANOVA post hoc Bonferroni test, CD: * p = 0.02, HFD: * p = 0.01, Control CD (n = 7), Aα4KO CD (n = 8), Control HFD (n = 9) and Aα4KO HFD (n = 8)) (**l**) Pancreas tissue sections in Control and Aα4KO mice fed a chow diet or HFD. Scale bars, 400 mm. (**m**) Tissue weights of Liver in Control and Aα4KO. Box plots are defined in terms of minima and maxima by whiskers, and the center and bounds of box by quartiles (One-way ANOVA post hoc Bonferroni test, ** p = 0.002, *** p < 0.0001, Control CD (n = 10), Aα4KO CD (n = 13), Control HFD (n = 11) and Aα4KO HFD (n = 9)). (**n**) Liver sections from Control and Aα4KO mice fed a chow diet or HFD stained with H&E (upper panel; Scale bars, 400 mm.) and Oil red O (bottom; Scale bars, 200 mm.). (**o**) mRNA levels of genes involved in gluconeogenic enzymes and inflammation after 12 weeks of the HFD (n = 10/group) or CD (n = 10/group). Box plots are defined in terms of minima and maxima by whiskers, and the center and bounds of box by quartiles (One-way ANOVA post hoc Bonferroni test, * p = 0.04, *** p < 0.0001).

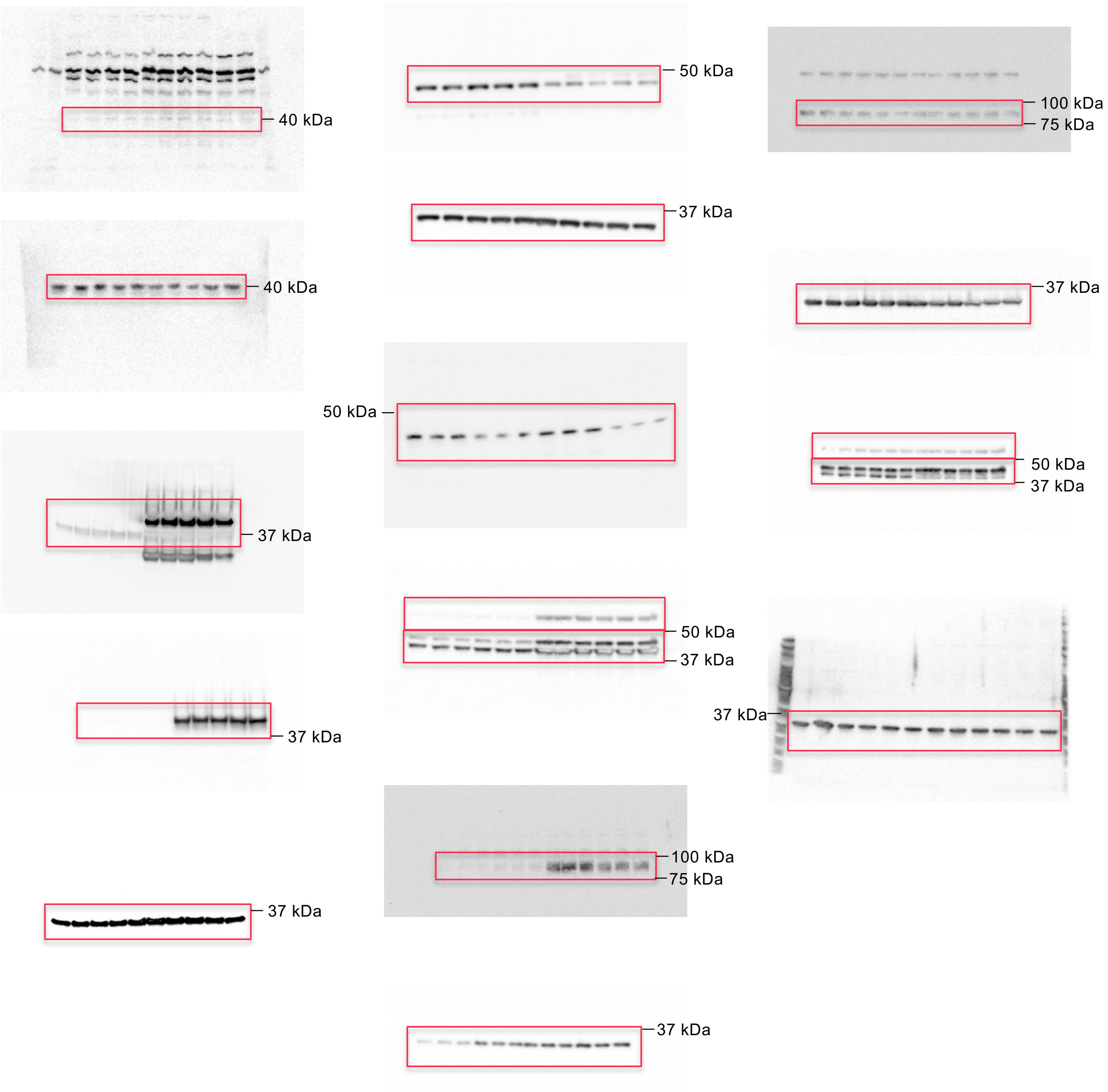

Supplementary Figure 8. Uncut blots. The red sections indicate blot results shown in Supplementary Figure 1.

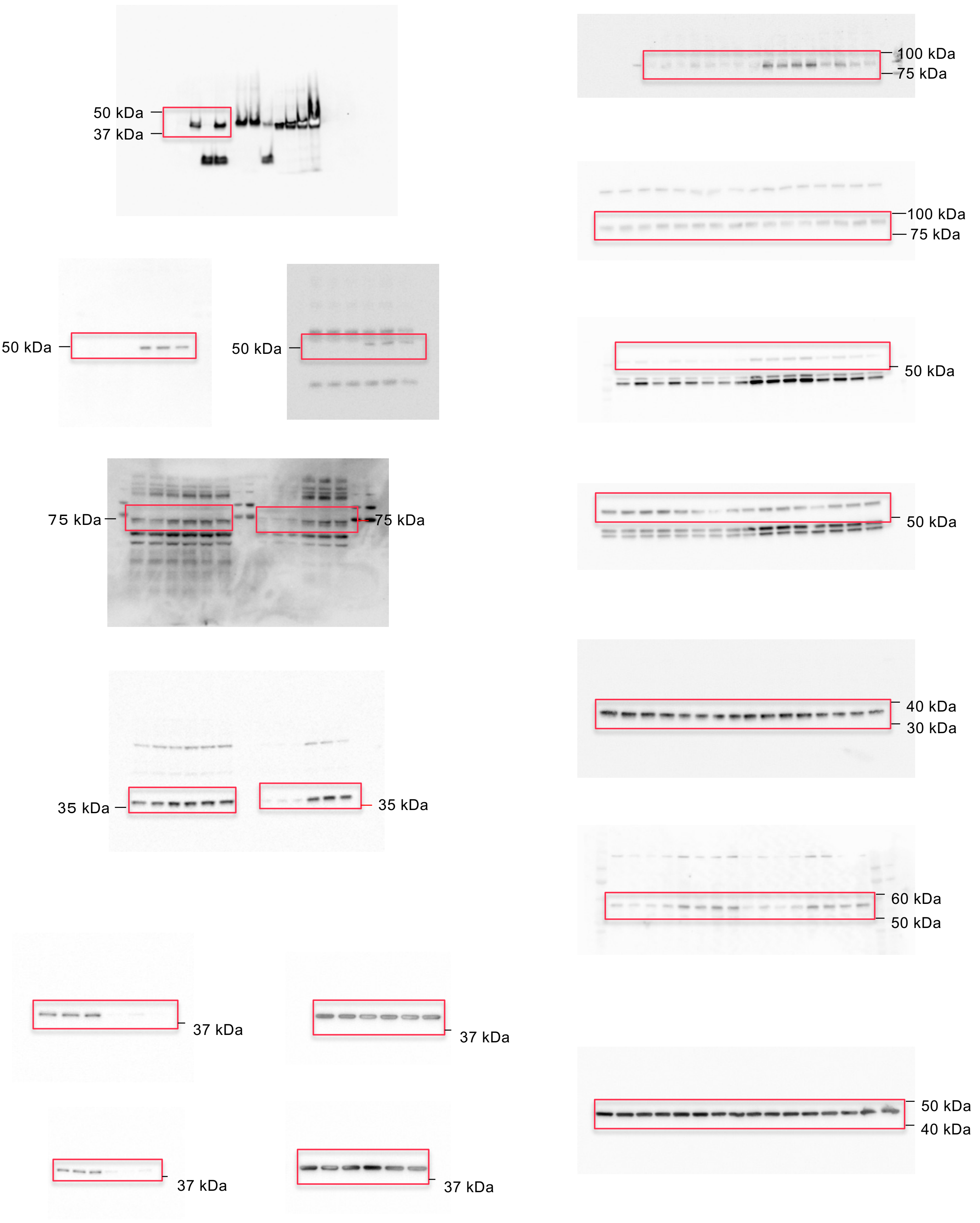

Supplementary Figure 9. Uncut blots. The red sections indicate blot results shown in Supplementary Figure 2 and Supplementary Figure3.

Supplementary Table 1. Quantitative Real-Time PCR primer sequences

Gene	Direction	Sequence	Gene	Direction	Sequence
TBP	Forward	ACCCTTCACCAATGACTCCTATG	h-UCP1	Forward	ACCGCAGGGAAAGAAACAGC
TBP	Reverse	TGACTGCAGCAAATCGCTTGG	h-UCP1	Reverse	TCAGATTGGGAGTAGTCCCT
FAS	Forward	GAGGACACTCAAGTGGCTGA	NFKB1	Forward	ATGGCAGACGATGATCCCTAC
FAS	Reverse	GTGAGGTTGCTGTCGTCTGT	NFKB1	Reverse	TGTTGACAGTGGTATTTCTGGTG
AP2	Forward	GATGCCTTTGTGGGAACCT	NFKB2	Forward	GGCCGGAAGACCTATCCTACT
AP2	Reverse	CTGTCGTCTGCGGTGATTT	NFKB2	Reverse	CTACAGACACAGCGCACACT
ATGL	Forward	ACTGTGGCCTCATTCCTCCT	Nlrp3	Forward	ATTACCCGCCCGAGAAAGG
ATGL	Reverse	AACTGGATGCTGGTGTTGGT	Nlrp3	Reverse	TCGCAGCAAAGATCCACACAG
Adiponectin	Forward	GATGGCACTCCTGGAGAGAA	PTP1B	Forward	GGAACTGGGCGGCTATTTACC
Adiponectin	Reverse	GCTTCTCCAGGCTCTCCTTT	PTP1B	Reverse	CAAAAGGGCTGACATCTCGGT
Leptin	Forward	GGGCTTCACCCCATTCTGA	Cxcl2	Forward	CCAACCACCAGGCTACAGG
Leptin	Reverse	TGGCTATCTGCACATTTTG	Cxcl2	Reverse	GCGTCACACTCAAGCTCTG
Glut4	Forward	ATCTTGATGACCGTGGCTCT	Cxcl10	Forward	CCAAGTGCTGCCGTCATTTTC
Glut4	Reverse	CTCAAAGAAGGCCACAAAGC	Cxcl10	Reverse	GGCTCGCAGGGATGATTTCAA
PPARγ	Forward	TGTTATGGGTGAAACTCTGGG	Cxcl12	Forward	TGCATCAGTGACGGTAAACCA
PPARγ	Reverse	AGAGCTGATTCCGAAGTTGG	Cxcl12	Reverse	TTCTTCAGCCGTGCAACAATC
Elovl3	Forward	GGACTTAAGGCCCTTTTTGG	Cxcl13	Forward	GGCCACGGTATTCTGGAAGC
Elovl3	Reverse	TTCCGCGTTCTCATGTAGGT	Cxcl13	Reverse	GGGCGTAACTTGAATCCGATCTA
Cidea	Forward	ATCACAACTGGCCTGGTTACG	Cxcl14	Forward	GAAGATGGTTATCGTCACCACC
Cidea	Reverse	TACTACCCGGTGTCCATTTCT	Cxcl14	Reverse	CGTTCCAGGCATTGTACCACT
Glut2	Forward	AGCTCCCTGGGATGAAGAG	Ccl3	Forward	TTCTCTGTACCATGACACTCTGC
Glut2	Reverse	ATCAAGAGGGCTCCAGTCAA	Ccl3	Reverse	CGTGGAATCTTCCGGCTGTAG
SREBP1c	Forward	ATCTCCTAGAGCGAGCGTTG	Ccl5	Forward	GCTGCTTTGCCTACCTCTCC
SREBP1c	Reverse	TATTTAGCAACTGCAGATATCCAA	Ccl5	Reverse	TCGAGTGACAAACACGACTGC
FBP1	Forward	CCATCATAATCGAACCTGAG	Ccl6	Forward	GCTGGCCTCATACAAGAAATGG
FBP1	Reverse	CTTCTCAGAAGGCTCATCAG	Ccl6	Reverse	GCTTAGGCACCTCTGAACTCTC
m-UCP1	Forward	ACTGCCACACCTCCAGTCATT	Ccl8	Forward	TCTACGCAGTGCTTCTTTGCC
m-UCP1	Reverse	CTTTGCCTCACTCAGGATTGG	Ccl8	Reverse	AAGGGGGATCTTCAGCTTTAGTA
Adrb3	Forward	GCTGACTTGGTAGTGGGACTC	Ccl9	Forward	CCCTCTCCTTCCTCATTCTTACA
Adrb3	Reverse	TAGAAGGAGACGGAGGAGGAG	Ccl9	Reverse	AGTCTTGAAAGCCCATGTGAAA
PRDM16	Forward	CAGCACGGTGAAGCCATTC	TNFa	Forward	ACGGCATGGATCTCAAAGAC
PRDM16	Reverse	GCGTGCATCCGCTTGTG	TNFa	Reverse	AGATAGCAAATCGGCTGACG
PGC1α	Forward	CCCTGCCATTGTTAAGACC	IL-6	Forward	TAGTCCTTCCTACCCCAATTTCC
PGC1α	Reverse	TGCTGCTGTTCCTGTTTTC	IL-6	Reverse	TTGGTCCTTAGCCACTCCTTC
CEBPα	Forward	CAAGAACAGCAACGAGTACCG	IL-1b	Forward	GCAACTGTTCCTGAACTCAACT
CEBPα	Reverse	GTCACTGGTCAACTCCAGCAC	IL-1b	Reverse	ATCTTTTGGGGTCCGTCAACT
Tfam	Forward	AGTTCCCACGCTGGTAGTGT	F4/80	Forward	CTGGGATCCTACAGCTGCTC
Tfam	Reverse	GCGCACATCTCGACCC	F4/80	Reverse	AGGAGCCTGGTACATTGGTG
Myd88	Forward	TCATGTTCTCCATACCCTTGGT	CD11c	Forward	CTGGATAGCCTTTCTTCTGCTG
Myd88	Reverse	AAACTGCGAGTGGGGTCAG	CD11c	Reverse	GCACACTGTGTCCGAACTCA
TLR4	Forward	ATGGCATGGCTTACACCACC	CCL2	Forward	TTAAAAACCTGGATCGGAACCAA
TLR4	Reverse	GAGGCCAATTTTGTCTCCACA	CCL2	Reverse	GCATTAGCTTCAGATTTACGGGT
Caspase1	Forward	ACAAGGCACGGGACCTATG	TGFb1	Forward	AAGTTGGCATGGTAGCCCTT
Caspase1	Reverse	TCCCAGTCAGTCCTGGAAATG	TGFb1	Reverse	GCCCTGGATACCAACTATTGC
IL-18	Forward	GACTCTTGCGTCAACTTCAAGG	Col1a1	Forward	CCTCAGGGTATTGCTGGACAAC
IL-18	Reverse	CAGGCTGTCTTTTGTCAACGA	Col1a1	Reverse	TTGATCCAGAAGGACCTTGTTTG

Supplementary Table 2. Quantitative Real-Time PCR primer sequences

Gene	Direction	Sequence	Gene	Direction	Sequence
Ndufs1	Forward	AGGATATGTTCGCACAACTGG	Ndufs1	Reverse	TCATGGTAACAGAATCGAGGGA
Ndufs2	Forward	CAGCCAGATATTGAATGGGCA	Ndufs2	Reverse	TGTTGGTCACCGCTTTTTCCT
Ndufs4	Forward	CTGCCGTTTCCGTCTGTAGAG	Ndufs4	Reverse	TGTTATTGCGAGCAGGAACAAA
Ndufs5	Forward	GACATACAGAAAAAGCTGGGCA	Ndufs5	Reverse	TCGCCTCATCGTTTTGTACCG
Ndufs7	Forward	GTTCATCAGAGTGTAGCCACTG	Ndufs7	Reverse	CAGGCCGAAGGTCATAGGC
Ndufs8	Forward	GTTCATAGGGTCAGAGGTCAAG	Ndufs8	Reverse	TCCATTAAGATGTCCTGTGCG
Cox5a	Forward	GCCGCTGTCTGTTCCATTC	Cox5a	Reverse	GCATCAATGTCTGGCTTGTTGAA
Cox5b	Forward	ACCCTAATCTAGTCCCGTCC	Cox5b	Reverse	CAGCCAAAACCAGATGACAG
Cox6c	Forward	GCGTCTGCGGGTTCATATTG	Cox6c	Reverse	TCTGCATACGCCTTCTTTCTTG
Cox6a1	Forward	TCAACGTGTTCCTCAAGTCGC	Cox6a1	Reverse	AGGGTATGGTTACCGTCTCCC
Cox7a1	Forward	GCTCTGGTCCGGTCTTTTAGC	Cox7a1	Reverse	GTACTGGGAGGTCATTGTCGG
Cox7a2	Forward	GCTGGCCCTTCGTCAGATT	Cox7a2	Reverse	GGCATCCCATTATCCTCCTGAA
Cox7b	Forward	TTGCCCTTAGCCAAAAACGC	Cox7b	Reverse	TCATGGAAACTAGGTGCCCTC
Cox8b	Forward	TGTGGGGATCTCAGCCATAGT	Cox8b	Reverse	AGTGGGCTAAGACCCATCCTG
NdufAB1	Forward	GGACCGAGTTCTGTATGTCTTG	NdufAB1	Reverse	AAACCCAAATTCGTCTTCCATG
