# Supplementary material for: Phosphatase protector alpha4 (α4) is involved in adipocyte maintenance and mitochondrial homeostasis through regulation of insulin signaling
Source: Nat Commun. 2022 Oct 14;13:6092. doi: 10.1038/s41467-022-33842-4 (PMC9568526; doi:10.1038/s41467-022-33842-4)
Supplement: Supplementary file 3 — Reporting Summary [file 41467_2022_33842_MOESM3_ESM.pdf]

nature portfolio

Corresponding author(s): Masaji Sakaguchi

Last updated by author(s): Sep 23, 2022

Reporting Summary

Nature Portfolio wishes to improve the reproducibility of the work that we publish. This form provides structure for consistency and transparency in reporting. For further information on Nature Portfolio policies, see our Editorial Policies and the Editorial Policy Checklist.

Statistics

For all statistical analyses, confirm that the following items are present in the figure legend, table legend, main text, or Methods section.

n/a	Confirmed	
☐	☒	The exact sample size (*n*) for each experimental group/condition, given as a discrete number and unit of measurement
☐	☒	A statement on whether measurements were taken from distinct samples or whether the same sample was measured repeatedly
☐	☒	The statistical test(s) used AND whether they are one- or two-sided<br>*Only common tests should be described solely by name; describe more complex techniques in the Methods section.*
☒	☐	A description of all covariates tested
☐	☒	A description of any assumptions or corrections, such as tests of normality and adjustment for multiple comparisons
☐	☒	A full description of the statistical parameters including central tendency (e.g. means) or other basic estimates (e.g. regression coefficient) AND variation (e.g. standard deviation) or associated estimates of uncertainty (e.g. confidence intervals)
☐	☒	For null hypothesis testing, the test statistic (e.g. *F*, *t*, *r*) with confidence intervals, effect sizes, degrees of freedom and *P* value noted<br>*Give P values as exact values whenever suitable.*
☒	☐	For Bayesian analysis, information on the choice of priors and Markov chain Monte Carlo settings
☒	☐	For hierarchical and complex designs, identification of the appropriate level for tests and full reporting of outcomes
☒	☐	Estimates of effect sizes (e.g. Cohen's *d*, Pearson's *r*), indicating how they were calculated

Our web collection on statistics for biologists contains articles on many of the points above.

Software and code

Policy information about availability of computer code

Data collection	Cooled CCD Camera System Light-Capture II (ATTO) was used to acquire western blot images. CS Analyzer version 2.0 (ATTO) was used to export and digitize western blot images.<br>Seahorse XFe24.<br>Multi-photon Laser Scanning Microscope (FV1000-MPE).<br>Confocal Laser Scanning Microscope (FV3000).<br>All-in-One Fluorescence Microscope (BZ-9000).<br>FLIR E53 Advanced Thermal Imaging Infrared Camera.<br>Implantable Temperature Transponders (IPTT-300).<br>In-vivo Micro-CT scanners (LaTheta™ LCT-100).<br>Cell Sorter SH800S (SONY).
Data analysis	Excel version 16.64.<br>Graph pad prism version 7.<br>File image J 1.52a.<br>BZ-II Dynamic Cell Count Analyzer (BZ-H1CE).<br>FlowJo™ v10 Software.<br>Adiposoft verion 1.16.

For manuscripts utilizing custom algorithms or software that are central to the research but not yet described in published literature, software must be made available to editors and reviewers. We strongly encourage code deposition in a community repository (e.g. GitHub). See the Nature Portfolio guidelines for submitting code & software for further information.

Data

Policy information about availability of data

All manuscripts must include a data availability statement. This statement should provide the following information, where applicable:

- Accession codes, unique identifiers, or web links for publicly available datasets
- A description of any restrictions on data availability
- For clinical datasets or third party data, please ensure that the statement adheres to our policy

The authors declare that the data supporting the findings of this study are available within the paper and its supplementary information/source data file. The raw data that support the findings of this study are available as source data file. RNA-seq data generated in this study are available at NCBI GEO database with the accession number GSE210407 and can be accessed with the following access link: [https://www.ncbi.nlm.nih.gov/geo/query/acc.cgi?acc=GSE210407]. Lipidomics raw data have been deposited via the figshare repository [https://doi.org/10.6084/m9.figshare.20417667]. All raw proteomic data have been deposited to the ProteomeXchange Consortium via the PRIDE partner repository with the dataset identifier PXD036915 [https://www.ebi.ac.uk/pride/archive/projects/PXD036915].

Human research participants

Policy information about studies involving human research participants and Sex and Gender in Research.

Reporting on sex and gender	This study did not involve human research participants.
Population characteristics	This study did not involve human research participants.
Recruitment	This study did not involve human research participants.
Ethics oversight	This study did not involve human research participants.

Note that full information on the approval of the study protocol must also be provided in the manuscript.

Field-specific reporting

Please select the one below that is the best fit for your research. If you are not sure, read the appropriate sections before making your selection.

☒ Life sciences ☐ Behavioural & social sciences ☐ Ecological, evolutionary & environmental sciences

For a reference copy of the document with all sections, see nature.com/documents/nr-reporting-summary-flat.pdf

Life sciences study design

All studies must disclose on these points even when the disclosure is negative.

Sample size	No statistical method was used to predetermine sample size. The cell-based experiments and the animal experiments, n=3-12 per group per experiment was chosen based on our previous experience with similar in vivo and in vitro experiments (PMID: 30952843 and PMID: 28065828).
Data exclusions	No data were excluded from the analysis.
Replication	The number of biological independent replicates indicated in the figure legends.
Randomization	The allocations of fluorescence images, H&E image analysis and EM images used this study were randomized by performing analysis at random positions. For in vivo studies, allocation of animals was randomly assigned to different groups, and all mice were matched for age and sex within the same experiment.
Blinding	Although the primary investigators were not originally blinded for most of the studies, the investigators performing the imaging analysis, flow cytometry and some of the sample analyses (such as measurement of blood parameters) were not informed of the expected results of the experiment before or during the analysis. Additionally, RNA-sequencing, proteomics and lipidomics were performed blinded.

Reporting for specific materials, systems and methods

We require information from authors about some types of materials, experimental systems and methods used in many studies. Here, indicate whether each material, system or method listed is relevant to your study. If you are not sure if a list item applies to your research, read the appropriate section before selecting a response.

Materials & experimental systems

n/a	Involved in the study	
☐	☒	Antibodies
☐	☒	Eukaryotic cell lines
☒	☐	Palaeontology and archaeology
☐	☒	Animals and other organisms
☒	☐	Clinical data
☒	☐	Dual use research of concern

Methods

n/a	Involved in the study	
☒	☐	ChIP-seq
☐	☒	Flow cytometry
☒	☐	MRI-based neuroimaging

Antibodies

Antibodies used

phospho-IR/IGF1R, 19H17, Cell Signaling Technology, (#3024, 1:1000)
IRβ, 4B8, Cell Signaling Technology, (#3025, 1:1000)
Phospho-p44/42 MAPK (Erk1/2) (Thr202/Tyr204), Cell Signaling Technology , (#9101, 1:1000)
p44/42 MAPK (Erk1/2), Cell Signaling Technology, (#9102, 1:1000)
phospho-Akt (S473), Cell Signaling Technology, (#9271, 1:1000)
Akt, 11E7, Cell Signaling Technology, (#4685, 1:1000)
phospho-S6 Ribosomal protein (S235/236), Cell Signaling Technology, (#2211, 1:2000)
S6 Ribosomal protein, 54D2, Cell Signaling Technology, (#2317, 1:1000)
phospho-YBX1 (S102), C34A2, Cell Signaling Technology, (#2900, 1:2000)
YB1, D299, Cell Signaling Technology, (#4202, 1:1000)
α4 (IGBP1), 5F6, Cell Signaling Technology, (#5699, 1:1000)
PP2A C Subunit, Cell Signaling Technology, (#2038, 1:1000)
GAPDH, D16H11, Cell Signaling Technology, (#5174, 1:1000)
beta-Actin, 13E5, Cell Signaling Technology, (#4970, 1:1000)
PTP1B, Abcam, [EPR22474] (ab244207, 1:1000)
Flag, Clone: M1, Sigma, (F3040, 1:5000)
Mid2, Abcam, (ab14749, 1:1000)
PP6 (E-2), Santa Cruz Biotechnology, (sc-393294, 1:1000)
CD45-PerCP-Cy5.5, Clone: 30F-11, BioLegend, (103131, 1:100)
F4/80-APC-Cy7, Clone: BM8, BioLegend, (BL123117, 1:100)
CD206-Alex647, Clone: MR5D3, Bio Rad, (MCA2235A647T, 1:50)
CD11c-PE, Clone: HL3, BD Pharmingen, (553802, 1:100)
Sytox Blue, Thermo Scientific, (S34857, 1:1000)
UCP1, Abcam, (ab10983, 1:100)
Perilipin A, Abcam, (ab61682, 1:100)
Cleaved Caspase3, Asp175, Cell Signaling Technology, (#9661, 1:100)
Iba-1, Wako, (019-19741, 1:200)
F4/80, Serotec, (MCA497R, 1:200)
Ki-67, Clone: MIB1, DAKO, (M7240, 1:100)
Insulin, Abcam, (ab7842, 1:500)
Anti-rabbit IgG, HRP-linked Antibody, Cell signaling, (#7074, 1:2000)
Anti-mouse IgG, HRP-linked Antibody, Cell signaling, (#7076, 1:2000)
mouse anti-goat IgG-HRP, Santa Cruz, (sc-2354, 1:1000)
Donkey anti-Rabbit IgG (H+L) Highly Cross-Adsorbed Secondary Antibody, Alexa Fluor Plus 594, Invitrogen, (A32754, 1:500)
Donkey anti-Goat IgG (H+L) Highly Cross-Adsorbed Secondary Antibody, Alexa Fluor Plus 488, Invitrogen, (A32814, 1:500)
Donkey anti-Mouse IgG (H+L), Alexa Fluor 488, Jackson ImmunoResearch, (715-546-150, 1:500)

Validation

The antibodies for phospho-IR/IGF1R, 19H17 (#3024, https://www.cellsignal.jp/products/primary-antibodies/phospho-igf-i-receptor-b-tyr1135-1136-insulin-receptor-b-tyr1150-1151-19h7-rabbit-mab/3024), IRβ, 4B8 (#3025, https://www.cellsignal.jp/products/primary-antibodies/insulin-receptor-b-4b8-rabbit-mab/3025), Phospho-p44/42 MAPK (Erk1/2) (Thr202/Tyr204) (#9101, https://www.cellsignal.jp/products/primary-antibodies/phospho-p44-42-mapk-erk1-2-thr202-tyr204-antibody/9101), p44/42 MAPK (Erk1/2) (#9102, https://www.cellsignal.jp/products/primary-antibodies/p44-42-mapk-erk1-2-antibody/9102), phospho-Akt (S473) (#9271, https://www.cellsignal.jp/products/primary-antibodies/phospho-akt-ser473-antibody/9271), Akt, 11E7, (#4685, https://www.cellsignal.jp/products/primary-antibodies/akt-pan-11e7-rabbit-mab/4685), phospho-S6 (S235/236)Ribosomal protein (#2211, https://www.cellsignal.jp/products/primary-antibodies/phospho-s6-ribosomal-protein-ser235-236-antibody/2211), S6 Ribosomal protein, 54D2,(#2317, https://www.cellsignal.jp/products/primary-antibodies/s6-ribosomal-protein-54d2-mouse-mab/2317) phospho-YBX1 (S102), C34A2, Cell Signaling Technology, (#2900, 1:2000), YB1, D299 (#4202, https://www.cellsignal.jp/products/primary-antibodies/phospho-yb1-ser102-c34a2-rabbit-mab/2900), α4 (IGBP1), 5F6 (#5699, https://en.cellsignal.jp/products/primary-antibodies/igbp1-5f6-mouse-mab/5699?Ns=product.displayName%7C0&N=102236+3390673522+4294956287&Nrpp=60&fromPage=plp), PP2A C Subunit (#2038, https://www.cellsignal.jp/products/primary-antibodies/pp2a-c-subunit-antibody/2038), GAPDH, D16H11 (#5174, https://www.cellsignal.jp/products/primary-antibodies/gapdh-d16h11-xp-rabbit-mab/5174), beta-Actin, 13E5 (#4970, https://www.cellsignal.jp/products/primary-antibodies/b-actin-13e5-rabbit-mab/4970) antibodies were validated for the western blotting for both human and mouse samples on the website of the Cell Signaling Technology. α4 (IGBP1) antibody was also detected by shRNA in mouse cell western blotting experiments. α4(IGBP1) antibody was validated using α4KO mouse cells and mice by the western blotting. PTP1B (ab244207, https://www.abcam.co.jp/ptp1b-antibody-epr22474-ab244207.html), Flag, Clone: M1,(F3040, https://www.sigmaaldrich.com/JP/ja/product/sigma/f3040) , Mid2 (ab14749, https://www.abcam.co.jp/mid2-antibody-ab14749.html), PP6 (E-2) (sc-393294, https://search.cosmobio.co.jp/view/p_view.asp?PrimaryKeyValue=7335650&ServerKey=&selPrice=1) antibodies were validated for the western blotting of mice samples on the websites of the companies. UCP1 (ab10983, https://www.abcam.co.jp/ucp1-antibody-ab10983.html), Perilipin A (ab61682, https://

www.abcam.co.jp/perilipin-1-antibody-ab61682.html), Cleaved Caspase3, Asp175 (#9661, https://www.cellsignal.jp/products/primary-antibodies/cleaved-caspase-3-asp175-antibody/9661), Iba-1 (019-19741, https://labchem-wako.fujifilm.com/jp/product/detail/W01W0101-1974.html), F4/80, Serotec, (MCA497R, https://bio-rad-antibody.jp/antibody/detail/gid:bio-rad/cnum:MCA497R/) Ki-67, Clone: MIB1 (M7240, https://www.labome.com/product/Dako/M7240.html) and Insulin (ab7842, https://www.abcam.com/insulin-antibody-ab7842.html) antibodies were validated for IHC studies of the mouse samples on the websites of the companies. CD45-PerCP-Cy5.5, Clone: 30F-11, (103131, https://www.biolegend.com/ja-jp/products/percp-cyanine5-5-anti-mouse-cd45-antibody-4264) and F4/80-APC-Cy7, Clone: BM8 (BL123117, https://www.biolegend.com/ja-jp/products/apc-cyanine7-anti-mouse-f4-80-antibody-4072) antibodies were validated for mice cells flow cytometry studies by BioLegend. CD206-Alex647, Clone: MR5D3, (MCA2235A647T, https://www.bio-rad-antibodies.com/monoclonal/mouse-cd206-antibody-mr5d3-mca2235.html?f=purified&JSESSIONID_STERLING=2B81DFA2ED6FB42EF54CAF521F5C5C24.ecommerce2&evCntryLang=JP-jathirdPartyCookieEnabled) and CD11c-PE, Clone: HL3, (553802, https://www.bdbiosciences.com/en-au/products/reagents/flow-cytometry-reagents/research-reagents/single-color-antibodies-ruo/pe-hamster-anti-mouse-cd11c.553802) antibodies were validated for mice cells flow cytometry studies on the websites of the companies. SYTOX™ Blue Dead Cell Stain, for flow cytometry was validated by Thermo Scientific (S34857, https://www.thermofisher.com/order/catalog/product/S34857).

Eukaryotic cell lines

Policy information about cell lines and Sex and Gender in Research

Cell line source(s)	Immortalized mouse brown preadipocytes (WT-1) were derived from the stromal vascular fraction (SVF) of interscapular brown adipose tissue of newborn mice. The SVF cells were immortalized by SV40 T overexpression as described in PMID: 14966273 and PMID: 18719589. HEK293 cells (human, RIKEN BRC, RCB-1637). Lenti-X 293T cells (Takara, cat. no. #632180). Immortalized human brown brown preadipocytes (A41 hBAT-SVF) were derived from the SVF of deep neck fat collected from a human subject. The SVF cells were immortalized by hTert overexpression as described in PMID: 26076036.
Authentication	The immortalized mouse brown preadipocytes (WT-1) have been deposited to Millipore Sigma and were authenticated by Millipore Sigma (#SCC255).<br>The immortalized human brown preadipocytes (A41 hBAT-SVF) have been deposited to ATCC and were authenticated by ATCC (#CRL-3385).
Mycoplasma contamination	Cell lines were screened for mycoplasma and they were negative for mycoplasma.
Commonly misidentified lines (See ICLAC register)	No commonly misidentified lines were used.

Animals and other research organisms

Policy information about studies involving animals; ARRIVE guidelines recommended for reporting animal research, and Sex and Gender in Research

Laboratory animals	Mice were housed at 20–22 °C on a 12 h-light/dark cycle with average 50% Humidity in the animal facility at Kumamoto University, Japan. All animal procedures were conducted according to the Guide for the Care and Use of Laboratory Animals issued by the Animal Research Committee at Kumamoto University (Kumamoto, Japan, Approval Numbers: A30-051 and 2020-099). Adiponectin-Cre mice (12-week-old male) were a generous gift from Evan D Rosen (Beth Israel Deaconess Medical Center and Harvard Medical School, Boston, USA) and can now be purchased at Jackson Laboratories (stock no. 010803). Adiponectin-CreERT2 mice (10-week-old, male, stock no. 025124) were purchased from Jackson Laboratories. For lineage tracing system of adipocytes Rosa-mTmG (10-week-old, male, Jax no. 007676) were purchased from Jackson Laboratories. Fat-specific conditional α4 KO (Aα4KO) mice were generated by breeding Adiponectin-CreER and α4 flox/flox mice (PMID: 11809737). Fat-specific inducible α4 KO (Ai-α4KO) mice were generated by breeding Adiponectin-CreERT2 and α4 flox/flox mice. Both Aα4KO and Ai-α4KO male mice age 8-20 weeks were used.
Wild animals	This study did not involve wild animals.
Reporting on sex	Male mice were used for all studies.
Field-collected samples	This study did not involve field-collected samples.
Ethics oversight	All animal procedures were conducted according to the Guide for the Care and Use of Laboratory Animals issued by the Animal Research Committee at Kumamoto University (Kumamoto, Japan, Approval Numbers: 2020-099).

Note that full information on the approval of the study protocol must also be provided in the manuscript.

Flow Cytometry

Plots

Confirm that:

☒ The axis labels state the marker and fluorochrome used (e.g. CD4-FITC).

☒ The axis scales are clearly visible. Include numbers along axes only for bottom left plot of group (a 'group' is an analysis of identical markers).

☒ All plots are contour plots with outliers or pseudocolor plots.

☒ A numerical value for number of cells or percentage (with statistics) is provided.

Methodology

Sample preparation	SVF was obtained from iWAT and BAT by treatment with 2 mg/ml collagenase (Sigma) for 45 min at 37 °C. The isolated SVF was resuspended in cold Hank's balanced salt solution (HBSS) with 2% fetal bovine serum (FBS). Cells were incubated with CD45-PerCP-Cy5.5 (BioLegend), F4/80-APC-Cy7 (BioLegend), CD206-Alex647 (Bio Rad) and CD11c-PE (BD Pharmingen) antibodies for 30 min in HBSS containing 2% FBS on ice and then washed and resuspended in solution with Sytox Blue (Thermo Scientific).
Instrument	Cell Sorter SH800S (SONY).
Software	FlowJo™ v10 Software.
Cell population abundance	There was no sorting performed. At least 100,000 cells were used for staining.
Gating strategy	FSC-A and SSC-A gates were adjusted to identify the cell populations. Doublets were excluded by the FSC-A and FSC-H scatter. The experiments analyzed a single cell population using one primary antibody. The dead cells were excluded by Sytox Blue staining. The SVF cells were gated on CD45+ cells and were further gated on a series of markers.

☒ Tick this box to confirm that a figure exemplifying the gating strategy is provided in the Supplementary Information.
